# Endoscopic Management of Benign Pancreaticobiliary Disorders

**DOI:** 10.3390/jcm14020494

**Published:** 2025-01-14

**Authors:** Amar Vedamurthy, Rajesh Krishnamoorthi, Shayan Irani, Richard Kozarek

**Affiliations:** 1 Division of Gastroenterology and Hepatology, Center for Digestive Health, Virginia Mason, Franciscan Health, Seattle, WA 98101, USA; 2 Center for Interventional Immunology, Benaroya Research Institute, Virginia Mason, Franciscan Health, 1100 9th Avenue, G-250B, Seattle, WA 98101, USA

**Keywords:** endoscopy, management, gallstones, cholecystitis, cholangitis, bile, leak, strictures, pancreatitis, necrosis, transplant, stents, calculi

## Abstract

Endoscopic management of benign pancreaticobiliary disorders encompasses a range of procedures designed to address complications in gallstone disease, choledocholithiasis, and pancreatic disorders. Acute cholecystitis is typically treated with cholecystectomy or percutaneous drainage (PT-GBD), but for high-risk or future surgical candidates, alternative decompression methods, such as endoscopic transpapillary gallbladder drainage (ETP-GBD), and endoscopic ultrasound (EUS)-guided gallbladder drainage (EUS-GBD), are effective. PT-GBD is associated with significant discomfort as well as variable adverse event rates. EUS-GBD leverages lumen-apposing metal stents (LAMS) for direct access to the gallbladder, providing the ability to treat an inflamed GB internally. Choledocholithiasis is primarily managed with ERCP, utilizing techniques to include balloon extraction, mechanical lithotripsy, or advanced methods such as electrohydraulic or laser lithotripsy in cases of complex stones. Altered anatomy from bariatric procedures like Roux-en-Y gastric bypass may necessitate specialized approaches, including balloon-assisted ERCP or EUS-directed transgastric ERCP (EDGE). Post-operative complications, including bile leaks and strictures, are managed endoscopically using sphincterotomy and stenting. Post-liver transplant anastomotic and non-anastomotic strictures often require repeated stent placements or advanced techniques like magnetic compression anastomosis in refractory cases. In chronic pancreatitis (CP), endoscopic approaches aim to relieve pain and address structural complications like pancreatic duct (PD) strictures and calculi. ERCP with sphincterotomy and stenting, along with extracorporeal shock wave lithotripsy (ESWL), achieves effective ductal clearance for PD stones. When traditional approaches are insufficient, direct visualization with peroral pancreatoscopy-assisted lithotripsy is utilized. EUS-guided interventions, such as cystgastrostomy, pancreaticogastrostomy, and celiac plexus blockade, offer alternative therapeutic options for pain management and drainage of peripancreatic fluid collections. EUS plays a diagnostic and therapeutic role in CP, with procedures tailored for high-risk patients or those with complex anatomy. As techniques evolve, endoscopic management provides minimally invasive alternatives for patients with complex benign pancreaticobiliary conditions, offering high clinical success and fewer complications.

## 1. Introduction

Benign pancreaticobiliary disorders, which include a variety of gallbladder, bile duct, and pancreatic conditions, are a significant source of morbidity worldwide. For instance, gallstone disease and its complications, including cholecystitis, choledocholithiasis, and acute pancreatitis account for a million emergency visits, 625,000 hospital discharges, and 2000 deaths annually [1]. Traditionally, these conditions have been managed with surgical interventions; however, many now benefit from endoscopic techniques that provide comparable outcomes with reduced invasiveness and faster recovery. Advances in endoscopic retrograde cholangiopancreatography (ERCP), endoscopic ultrasound (EUS)-guided interventions, and stent technologies have broadened the therapeutic options available. These innovations are instrumental in addressing challenges ranging from acute cholecystitis to complications related to the bile duct and pancreatic duct. They are especially valuable for patients with complex or altered anatomy, as well as for those who cannot undergo surgery [2]. This narrative review provides an overview of the endoscopic procedures available for managing benign pancreaticobiliary diseases. It places particular emphasis on the endoscopic management of acute cholecystitis, bile duct disorders in patients with both normal and variant anatomy, and complications associated with acute and chronic pancreatitis. While it offers a broad perspective for general practitioners, the manuscript also aims to serve as a focused resource for gastroenterologists and surgeons looking to integrate advanced endoscopic techniques into their practice.

## 2. Methods

This narrative review examines the literature on benign pancreaticobiliary disorders treated with endoscopic therapies. We conducted searches on PubMed and Google Scholar to identify relevant articles using titles or keywords such as “Cholecystitis”, “Choledocholithiasis”, “Post-hepatobiliary surgery complications”, “Bile leak”, “Biliary stricture”, “post-liver transplant biliary strictures”, “Recurrent acute pancreatitis”, “Pancreatic fluid collections”, and “Endoscopic ultrasound directed therapies”. Articles in languages other than English or those lacking full text availability were excluded. Systematic reviews, meta-analyses, and randomized controlled trials were prioritized for inclusion. Ultimately, a total of 80 articles were included in the final review.

## 3. Discussion

### 3.1. Gallstone Disease

Gallstones are the commonest cause of emergency room presentation in patients with abdominal pain. Asymptomatic gallstones do not need treatment. In symptomatic patients, they can result in complications as a consequence of cystic or common bile duct blockage.

### 3.2. Acute Cholecystitis

Acute cholecystitis is caused by the obstruction of the cystic duct, leading to inflammation of the gallbladder (GB). The diagnosis is typically made with US or CT, which shows pericholecystic fluid collection, wall thickening, or distension. Cholecystectomy remains the standard of care. In cases where surgery is deemed high risk, decompression should be considered to prevent complications such as perforation, gangrene, or fistulization into the bowel. The most common technique for decompression is percutaneous drainage (PT-GBD), along with antibiotic therapy. PT-GBD can be cumbersome to manage due to the presence of an external drain and a drainage bag. The availability of procedural expertise and recommendations from extensive studies led to the rise in the use of PT-GBD. Multicenter studies have shown that PT-GBD is associated with higher post-procedure pain scores and high adverse events at 30 days and 1 year [3,4,5]. The external catheter must remain in place until the tract matures and becomes potentially longer. There are two endoscopic treatment options available for the management of acute cholecystitis, specifically in patients with high co-morbidities or deemed unfit for surgery. Decompression can be performed in one of two ways: internal drainage can be performed by placing a transpapillary stent into the gallbladder; or lumen through ERCP or transmurally by placement of a metal stent from the stomach or the duodenum.

### 3.3. Transpapillary Gallbladder Drainage (ETP-GBD)

**Figure 1:**
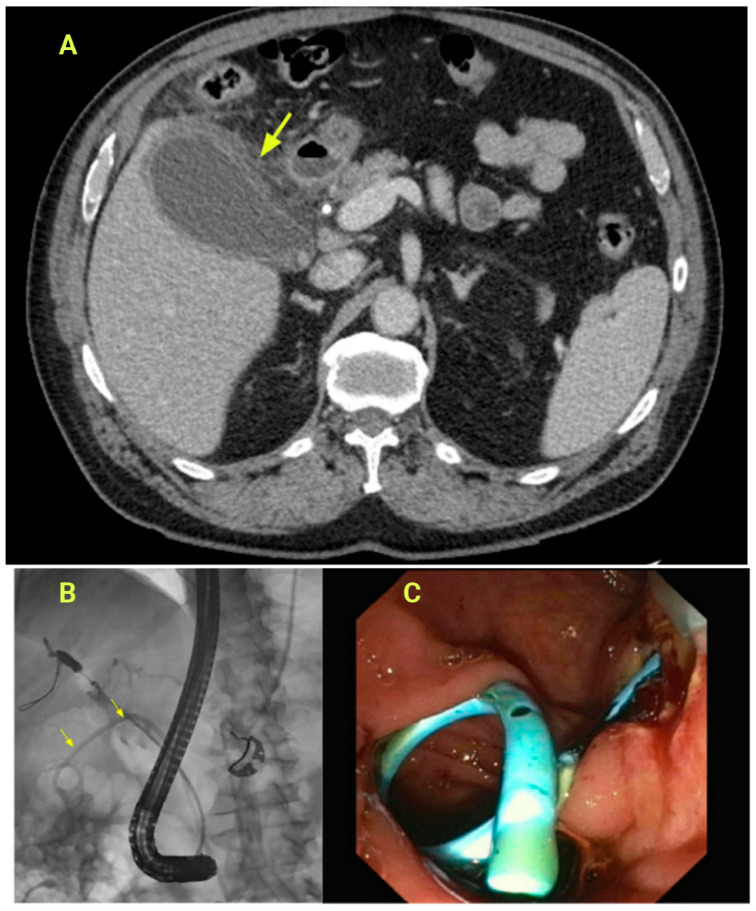
(**A**): CT scan demonstrating gallbladder wall thickening with pericholecystic fluid. The patient was on anticoagulation at the time of presentation (**Top**). Arrow shows thickened gallbladder wall with pericholecystic fluid. (**B**): ERCP with the placement of transpapillary double pigtail stent for acute cholecystitis (**Left**). Arrows demonstrated the pigtail of the stent within the gallbladder lumen. (**C**): Endoscopic view of transpapillary gallbladder stent placement (**Right**).

The technique was first described by Kozarek in 1984 [6]. This involves the following: deep bile duct cannulation using ERCP techniques, visualization of the cystic duct by cholangiography, the passage of a hydrophilic guidewire in the cystic duct to curl in the gallbladder and insertion of a long 7 Fr double pigtail stent, leaving one pigtail in the gallbladder and the other in the duodenum (refer to Figure 1). Endoscopic transpapillary GB drainage is an effective procedure in patients with ascites, severe coagulopathy, and inaccessible gallbladder with EUS. It can be technically challenging due to the tortuous nature of the cyst duct and whether the cystic duct has inflammatory changes from the extension of gallbladder inflammation. Cystic duct obstruction and the inability to identify the cystic duct on cholangiography are additional challenges. Special designs of stents have also been developed specifically for ETP-GBD. Glessing et al. described the use of a Johlin pancreatic wedge stent compared to a standard pigtail [7]. Some case series have reported the use of direct cholangioscopy to identify the cystic duct take-off and guide the placement of a wire into the gallbladder. However, cannulating the cystic duct with ERCP can be challenging, and there is a risk of perforation during attempts to pass the guidewire [8,9]. A sphincterotomy may be advanced deep into the bile duct and withdrawn slowly; the tip may catch the cystic duct opening [10]. Transpapillary GB drainage is an important option for patients with higher comorbidities. This technique can also lead to a potential surgical option should the patient’s condition improve. The surgeon simply removes the stent from the gallbladder at the time of cholecystectomy. In the series by Irani et al., most transpapillary drainage cases proceeded to cholecystectomy. Patients with ascites have difficulty with tract maturation. Some experts recommend performing a small-volume paracentesis prior to the attempt. Significant ascites lead to inadequate apposition of the GB to the luminal tract (EUS-GBD) or skin (PT-GBD) which could result in bile leak. Hence, these patients should be considered for transpapillary drainage before attempts at EUS-guided drainage. Kjaer et al. [11] showed that technical success improves with experience, demonstrating an improvement from 50% to 89% after 4–5 years of adopting the technique. An international multicenter study comparing transpapillary versus EUS versus percutaneous (PCT) drainage found significantly lower technical and clinical success rates in the ETP-GBD [12]. Reported adverse events of this technique include cystic duct perforation, cholangitis, and sepsis, as well as the inherent risks of ERCP, such as post-ERCP pancreatitis (PEP) and sphincterotomy-related bleeding (PSB). Refer to Table 1 for a summary of outcomes associated with ETP-GBD.

### 3.4. Endoscopic Ultrasound-Guided Gallbladder Drainage (EUS-GBD)

**Figure 2:**
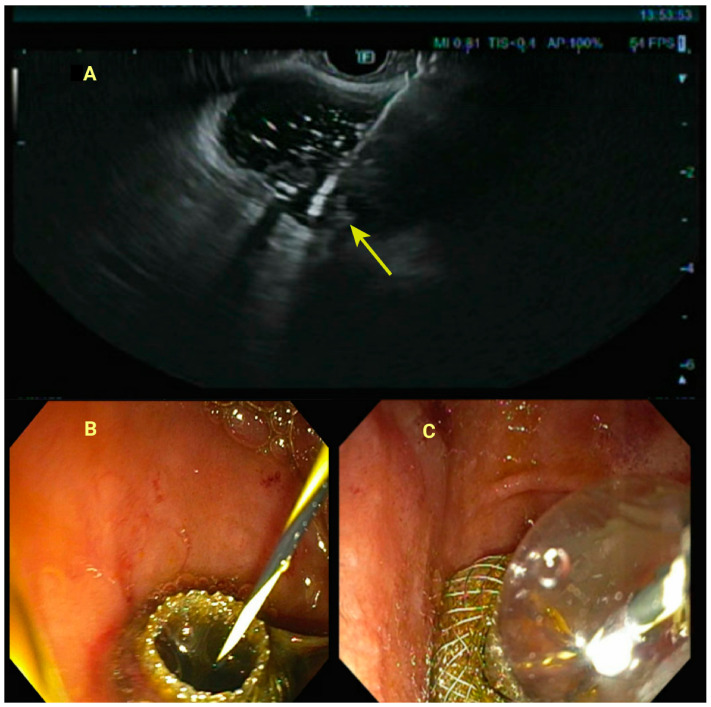
(**A**): Deployment of distal flange (yellow arrow of the lumen-apposing metal stent into the gallbladder lumen). (**B**): View of the lumen-apposing metal stent from the duodenum. (**C**): Balloon dilation of the lumen-apposing metal stent to allow drainage of bile.

The second technique of gallbladder decompression includes the use of endoscopic ultrasound (EUS). Initial attempts with EUS drainage included the use of a 19G needle to puncture the gastric or duodenal wall to enter the gallbladder lumen. A guidewire is then coiled within. The tract is then serially dilated with a graded dilation catheter or a dilating balloon. The procedure was initially performed using 7 Fr pigtail stents or biliary fully covered metal stents. These were prone to migration. The technique has evolved since the advent of lumen-apposing metal stents (LAMS). Refer to Figure 2. LAMS are available in different sizes and are fully covered. A 10 mm × 10 mm LAMS is usually the preferred size for gallbladder drainage. A LAMS is dumbbell-shaped, deployed under EUS guidance, and the flanges hold the layers of the gallbladder wall and gastrointestinal lumen in apposition, thereby preventing bile leak. The procedure involves the insertion of a LAMS between the gallbladder and the stomach or duodenum [16]. EUS-GBD has very high technical and clinical success rates ranging from 90% to 98% and 89 to 98.4% in expert hands. Non-surgical candidates can be treated endoscopically with EUS-guided gallbladder drainage. This method avoids the risk of PEP. Secondly, it provides access to the gallbladder lumen for treatment of gallstones using lithotripsy. This technique is technically not feasible in patients with ascites due to the risk of bile leak and inability to form a fistulous tract.

**Figure 3:**
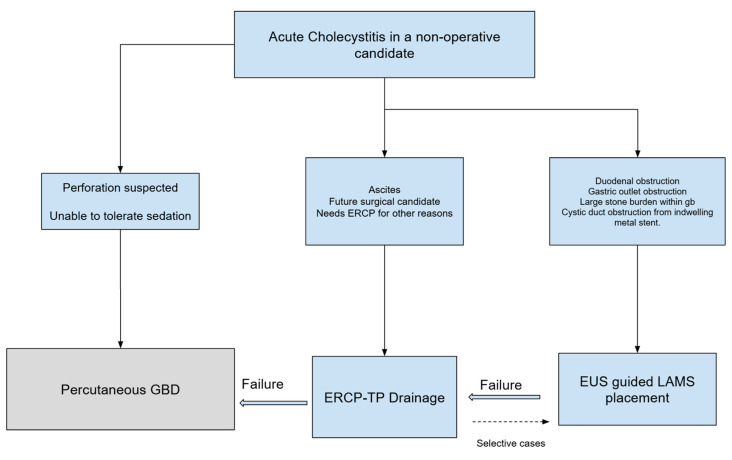
Suggested algorithm for endoscopic management of acute cholecystitis.

The creation of a cholecystoduodenal or cholecystogastric fistula may affect future candidacy for surgery. A multicenter international study [17] showed a shorter surgical procedure, shorter length of stay, and shorter time to cholecystectomy when compared to PT-GBD. The operative adverse events were similar between the PT-GBD and EUS-GBD groups. However, the rate of open conversion was higher in the EUS-GBD but failed to reach statistical significance [18] and reported three cases of failed robotic or laparoscopic cholecystectomy due to the presence of adhesions and fistula. See Figure 3 for a suggested algorithm for endoscopic management of acute cholecystitis.

Compared to PCT, there is no inconvenience or discomfort in maintaining an external drain. The LAMS will often allow complete stone clearance. Once the gallbladder is cleared, exchanging the LAMS for plastic pigtail stents helps to maintain the cholecystointestinal fistula and potentially reduce the risks of an indwelling LAMS. Percutaneous drainage serves as a bridge to future cholecystectomy. However, in patients who were deemed fit for surgery due to acute illnesses precluding surgical options, resulting in long-term external GB drainage, conversion to internal EUS-GBD can be considered. A study by Law et al. [19] suggests that internalization of PT-GBD catheters using EUS GBD and LAMS placement as a conversion strategy is feasible and was performed successfully in seven patients without adverse events. In transmural drainage with EUS-GBD, there is the risk of bile leak or gallbladder perforation from stent misdeployment, bleeding, and occlusion of stents by food material. However, adverse event rates are similar to percutaneous drains placed by interventional Radiology. See Table 2 for procedural outcomes of EUS-GBD.

### 3.5. Choledocholithiasis

Choledocholithiasis refers to the presence of stones within the extrahepatic biliary system. It can be primary or secondary. Secondary stones originate within the gallbladder and migrate into the CBD via the cystic duct. The diagnosis is based on abnormal bilirubin, alkaline phosphatase, or GGT. The biochemical tests progressively increase with the duration and severity of biliary obstruction. Transabdominal US has a relatively poor sensitivity (22–55%) for detecting CBD stones but a higher sensitivity for CBD dilation (77–87%) [21]. Helical CT has shown a sensitivity of 88% and specificity of 97%. MR has 85–92% sensitivity and 93–97% specificity for choledocholithiasis detection. However, the sensitivity of MRCP seems to diminish in the setting of small (<6 mm) stones, and sensitivity is reduced to 33–71% in this subset [22]. Intraoperative cholangiography [IOC] is performed in the setting of cholecystectomy and can be successfully done in 88–100% of patients. A sensitivity of 59–100% and specificity of 93–199% for choledocholithiasis has been reported. It requires an additional 10–15 min during a laparoscopic cholecystectomy [23]. EUS is the most sensitive test for the detection of choledocholithiasis, with a sensitivity greater than 95% and 100% specificity [23,24].

### 3.6. Endoscopic Retrograde Cholangiopancreatography (ERCP)

**Figure 4:**
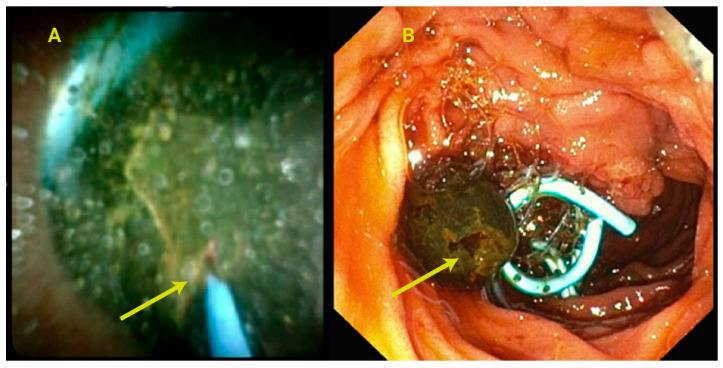
(**A**): A large, impacted stone leading to multiple failed attempts with extraction balloon, mechanical lithotripsy; stone was fragmented using a laser lithotripter (yellow arrow) and could be removed with extraction balloon after fragmentation. (**B**): Shows the effect of laser lithotriptor on the stone. A plastic double pigtail stent is placed in the bile duct to prevent cholangitis from impaction of stone fragments.

The majority of patients with CBD stones are treated with biliary sphincterotomy and an extraction balloon or a retrieval basket. This technique works for the majority of stones smaller than 10 mm. With larger stones, dilation-assisted stone extraction can be performed (DASE). The latter is the combination of two techniques: endoscopic papillary large balloon dilation and a limited sphincterotomy. In some cases of large, irregularly shaped, or boxcar stones, mechanical lithotripsy and cholangioscopy-guided techniques such as electrohydraulic lithotripsy or laser lithotripsy can be performed (see Figure 4). A meta-analysis concluded that biliary sphincterotomy plus balloon sphincteroplasty was associated with fewer complications than biliary sphincterotomy alone [25]. Mechanical lithotripsy involves the use of a cranking mechanism attached to a stone retrieval basket. Single operator cholangioscopy with laser lithotripsy or EHL has been reported to have a stone clearance of 88%, with some reports reaching 100% with repeated sessions. Because of cost issues, many endoscopists employ DASE or mechanical lithotripsy prior to resorting to SOC. However, in a network meta-analysis, SOC resulted in statistically significantly higher rates of complete stone removal than mechanical lithotripsy. (RR of 1.5, *p* < 0.04). EHL systems work through a bipolar probe and charge generator. The tip of the probe generates a spark, leading to the expansion of surrounding fluid, creating an oscillating shock wave of pressure that fragments the stone [26]. LL works by focusing laser light of a high-power density on the surface of a stone. The concentrated high power creates a plasma composed of a gaseous collection of ions and free electrons that oscillates and induces waves breaking the stone. EHL equipment is relatively inexpensive compared to LL. However, the risk of perforation is higher in EHL than in LL [26].

In a systematic review and meta-analysis, LL had a higher rate of ductal clearance (95%) than EHL (75.5%) and ESWL (89.3%). The post-procedure complication rates were higher for patients treated with EHL (13%) compared to ESWL (8.4%) or LL (9.6%) [27]. Anecdotally, plastic biliary stent placement alone can fragment residual stones over time, making subsequent stone extraction easier.

### 3.7. Altered Anatomy

**Figure 5:**
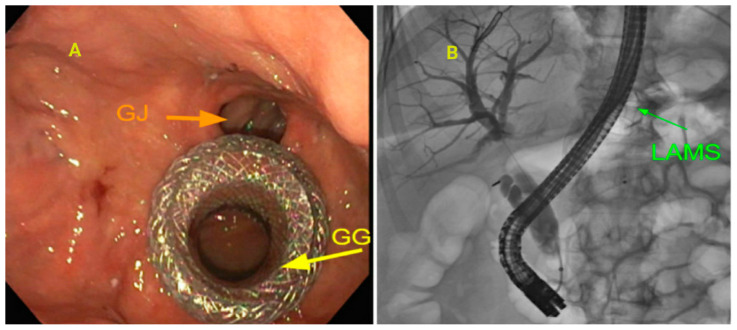
(**A**): Patient with RYGB gastric bypass, presents with choledocholithiasis, undergoes EDGE procedure to enable ERCP through the excluded stomach. GG—gastrogastrostomy, GJ—gastrojejunostomy. LAMS—lumen-apposing metal stent. (**B**): Shows the passage of duodenoscope through the LAMS into the descending duodenum to perform ERCP.

**Figure 6:**
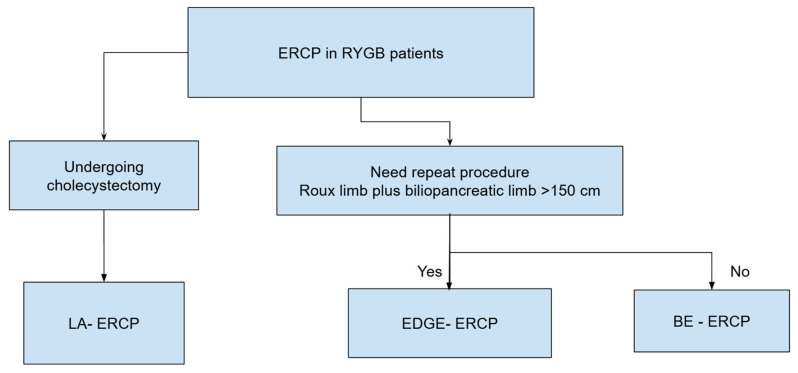
Suggested algorithm for endoscopic management of choledocholithiasis in Roux-en-Y gastric bypass patients.

Roux-en-Y gastric bypass (RYGB) is one of the most common bariatric procedures worldwide. The altered anatomy requires traversing a significant length of the alimentary and biliopancreatic limbs to reach the papilla. This led to the development of balloon-assisted ERCP (BE-ERCP). The use of a single balloon or double balloon-assisted enteroscope facilitates the ability to reach the papilla and perform biliary interventions. A multicenter study of ERCPs performed using a short DBE in 39 RYGB patients noted an 82% success rate for reaching the papilla and a 90% cannulation rate. The complication rate was 5%. However, BE ERCP can be challenging due to the following factors: the absence of an elevator, using a forward viewing scope, and limited accessories. In failed cases, we can resort to laparoscopic-assisted ERCP (LA ERCP). However, LA ERCP is associated with longer hospital stays, an increase in costs, higher complications, and the need to involve both surgery and interventional endoscopy teams. Endoscopic ultrasound-directed transgastric ERCP (EDGE) was first described by Kedia et al. [28]. EDGE-ERCP uses a LAMS to create agastrogastrostomy or gastrojejunostomy connecting the gastric pouch or the proximal jejunum to the excluded stomach. See Figure 5. This enables the usage of a duodenoscope to perform the ERCP [29]. Based on a meta-analysis, the pooled rate of technical success of EDGE in RYGB was similar to LA-ERCP and was superior to BE ERCP. The most commonly reported adverse event is the failure of gastro-gastric fistula closure, requiring the need for over-the-scope clips or endoscopic suturing. See Figure 6 for a suggested algorithm in the management of choledocholithiasis in RYGB patients.

### 3.8. Post-Operative Complications

Bile leaks most commonly occur in the setting of severe acute cholecystitis and often are associated with incomplete cholecystectomy. Likewise, loss of tissue planes from severe cholecystitis or an intrahepatic gall bladder may result in inadvertent and unrecognized laceration, transection, or clip placement on the common bile duct, the latter sometimes presenting months or even years later with jaundice, elevated liver functions, or stone formation proximal to the clip.

### 3.9. Bile Leak and Strictures

Bile leaks continue to be a persistent challenge following liver and biliary tract surgeries. Post cholecystectomy, bile leaks occur in 0.3–2.7% of patients, and post-hepatectomy leaks range from 3–20%. The risk factors for the probability of a bile leak after surgery include choledocholithiasis, gangrenous cholecystitis, or severe inflammation, which makes the surgery complex. Cystic duct stump leakage occurs from misplaced or displaced clipping, electrosurgical injury to the cystic duct, or from excessive traction. More commonly, the impaction of a stone in the ampulla leads to dehiscence of the cystic duct stump. Ducts of Luschka can also be severed, leading to a bile leak. The first line for management of biliary leaks has been the endoscopic approach using ERCP. Sandha et al. classified post-lap-cholecystectomy (LC) bile leak into low-grade and high-grade. Low-grade leaks are identified after intrahepatic opacification, and high-grade leaks occur before intrahepatic opacification. The study reported a 91% success rate with biliary sphincterotomy alone with low-grade leaks and 100% in high-grade leaks when stents were used. High-grade bile leaks occur due to a tear in the CBD wall or one of the biliary branches that form the main hepatic confluence. A stent placement benefits this in two ways: to reduce the transpapillary pressure and bypass the leak, thereby diverting bile flow into the duodenum. Stent placement also prevents secondary stricture formation at the site of injury. The stents are left in place for a minimum of 4 weeks. The success rate of endoscopic therapy remains high. The treatment aims to reduce ampullary resistance, thereby facilitating transpapillary bile flow and reducing leakage. During ERCP, there are options for sphincterotomy with or without stent placement. Sphincterotomy alone could be considered in the presence of choledocholithiasis, which causes a small leak. Sphincterotomy, in addition to plastic or fully covered metal stents, can be considered for high-grade or complex leaks. The use of multiple plastic stents or fully covered metal stents can be considered in refractory leaks [30,31,32,33].

Bergman et al. classified bile duct injuries into four categories [34]: A—cystic duct leak from aberrant or hepatic radicles (minor lesions), B—major bile duct leaks with or without concomitant strictures, C—bile duct strictures without leakage, and D—Complete transection of the duct with or without excision of a portion of the biliary tree. Post-operative bile duct strictures occur in 0.2–0.5% of patients following LC [35]. They are often due to direct surgical trauma from partial or complete transection by clipping or ligation of the bile duct. The stricture can also occur due to an ischemic insult to the biliary tree during dissection or cauterization [36]. The outcome of endotherapy depends on factors such as early identification and referral. Only 10% of post-operative strictures are identified within the first week after LC, and nearly 70% within the first six months [37]. Occasionally, the diagnosis can be delayed for several years. Such patients present with cholangitis or choledocholithiasis. The endoscopic approach remains the first line for the management of post-operative strictures.

**Figure 7:**
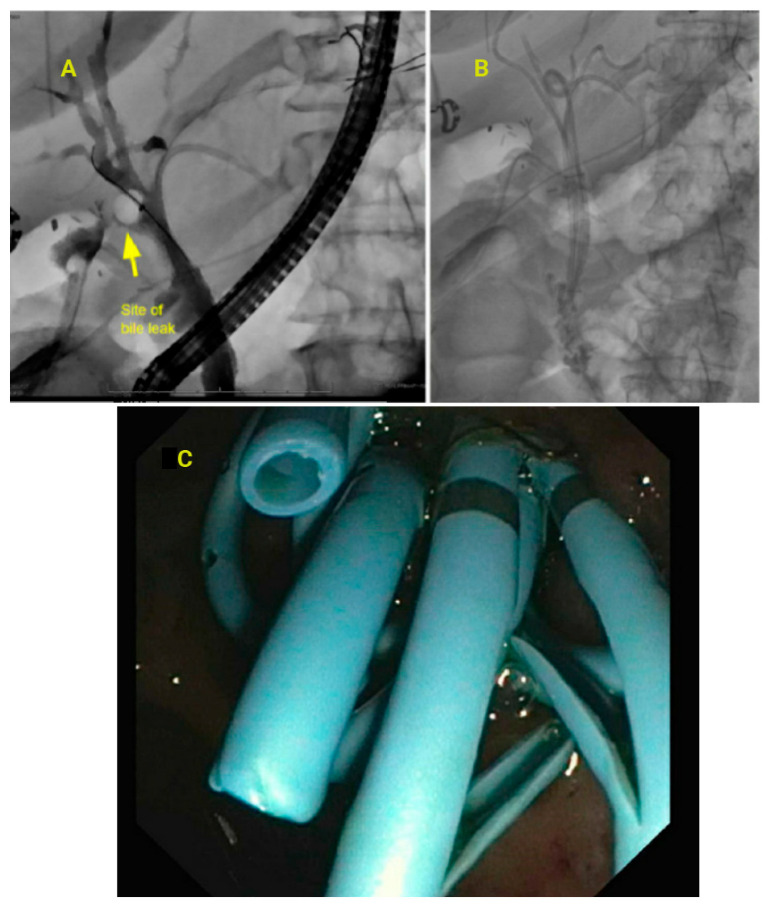
(**A**): High-grade bile leak noted at the common hepatic duct, treated with multiple plastic stents draining different segments, diverting bile from the site of leak. (**B**): High-grade bile leak treated with multiple double pigtail plastic stents (**C**): Endoscopic view of the transpapillary plastic stents.

Endotherapy, albeit requiring multiple sessions, is often successful except in certain cases of complete transection of the bile duct. Plastic stents of 10–12 Fr placement/replacement every 3–4 months, along with balloon dilation for a period of 12 months, are associated with the disappearance of stricture [38] Costamagna et al. showed that multiple stent placement resulted in stricture resolution in 97% of cases, with a recurrence rate of 9.4% after a mean follow-up of 11.2 years. Patients who received more than four plastic stents had a reduced recurrence rate, supporting the need for an aggressive approach. A randomized controlled trial with 31 patients compared multiple plastic stents (MPS) (>4) versus partially covered SEMS (PCSEMS) (8–10 mm) to treat post-surgery biliary strictures showed a higher adverse event in the MPS group compared with PCSEMS [39]. See Figure 7 for a case of high-grade bile leak treated with multiple plastic stents. Biliary patency was higher in the PCSEMS group. In LC patients, the biliary strictures are closer to the hepatic hilum [40]; hence, placement of SEMS is technically challenging due to the risk of side branch occlusion. The use of an FCSEMS for post-cholecystectomy bile duct strictures is currently off-label [41]. A recently published study by [42] Jang et al. showed that magnetic compression devices can be used for complete biliary obstruction post LC. The recent advances in endoscopic treatment with stents for the treatment of major bile duct injuries and strictures are at least as effective as surgical treatment. However, endoscopic management and surgical therapies are complementary and should be considered on a case-by-case basis based on complexity.

### 3.10. Post-Liver Transplant (LT) Biliary Strictures

Post-transplant biliary strictures are classified as anastomotic and non-anastomotic. Most centers perform a biliary duct-to-duct anastomosis at the time of liver transplant, as it allows access for future endoscopic interventions and retains the sphincter functions. Anastomotic biliary strictures are known to be more common in living donor LT than in deceased donor LT, with rates similar to a Roux-en Y choledochojejunostomy. Bile leaks post LT occur in the first month and mostly at the anastomotic site [43]. Bile leaks post LT are treated similarly to post-LC leaks. Plastic biliary stents (10–11.5 Fr), with or without biliary sphincterotomy placed across the leak, have a success rate of 90–95% [44]. However, the stents are left in place for 6–8 weeks due to the delayed healing that occurs as a consequence of immunosuppression [45,46].

In a systematic review of 200 patients by Kao et al., structures associated with LT resolved after placement of multiple plastic stents at 12 months (94–100%), comparable to resolution rates of 80–95% for SEMS left in place for 3 months or longer. However, the overall migration rate was 16% in the SEMS group [47]. Plastic stent distal migration occurs in 5–10% of cases in single stent placement compared to multiple stent placement. The steps involved in the management of an anastomotic stricture start by traversing the stricture with a guidewire. This maneuver is more challenging in post-operative strictures than malignant strictures. The narrow and often angulated lumen is cannulated with hydrophilic guidewires measuring 0.021 or 0.018 inches with a straight or angled tip. Secondly, dilation of the stricture using a hydrostatic balloon facilitates stent placement. Lastly, stent placement keeps the stricture open for an extended period, leading to remodeling. The majority of anastomotic strictures occur within the first year after liver transplantation. A thin narrowing occurs in the area of biliary anastomosis, which becomes apparent after the resolution of post-operative edema and inflammation. Biliary strictures occurring at or greater than 5 mm proximal to the anastomosis are considered non-anastomotic (NAS) biliary strictures caused by ischemia secondary to hepatic artery stenosis or thrombosis. This stricture mostly occurs at the hilum but can also involve the intrahepatic ducts. Endoscopic therapy is not as effective for NAS, with an estimated success rate of 40–82% [48,49]. Such strictures often require repeated dilations and prolonged stenting [50]

Newer devices, such as magnetic compression anastomosis, are available in certain centers to treat anastomotic strictures refractory to conventional procedures. Two tracts are required to deliver the magnets above and below the strictures. This can lead to new fistula formation and is similar to a surgical anastomosis [51]. After 6 weeks, the magnets are removed, and an FCSEMS is placed for 6 months. This technique, however, lacks long-term study data and clinical trials to support routine use. Refer to Table 3 for procedural outcomes of anastomotic strictures treated with plastic stents.

### 3.11. Benign Pancreatic Disorders

There are myriad benign pancreatic disorders for which EUS and ERCP are used, to include defining the characteristics of pancreatic cysts and strictures to assure that they are benign. Therapeutically these techniques have been integrated into endoscopic practice in pancreaticobiliary referral centers. Although not an exhaustive list, the following are the most common applications of endoscopic therapies currently used.

### 3.12. Recurrent Acute Pancreatitis

Recurrent acute pancreatitis (RAP) is defined as two or more episodes of acute pancreatitis (AP) with clinical and radiologic remission between episodes. The term idiopathic RAP is used when the cause remains unidentified based on history, exam, biochemical tests, and imaging. Common causes include biliary microlithiasis caused by rapid weight loss, critical illness, and in organ transplant recipients. Stones < 3 mm that are not visible on routine imaging impact at the ampulla of Vater leading to inflammation and fibrosis. This results in ampullary stenosis thereby causing biliary pancreatitis. Historically, experts have resorted to empiric cholecystectomy even if there is an insignificant stone burden or sludge. ERCP with ES is an effective alternative treatment, as it allows crystals and small stones to pass without causing pancreatic duct obstructions. Pancreatic divisum is the nonunion of the ventral and dorsal ducts resulting in the majority of drainage occurring through the minor papilla. This can be a cause of RAP. However, the decision to intervene should be carefully assessed. Multiple observational studies have shown the benefit of minor papillotomy or transpapillary dilation in reducing attacks of pancreatitis. The aim of this therapy in symptomatic patients with pancreatic divisum is to relieve outflow obstruction at the level of minor papilla. A randomized controlled clinical trial shows patients with minor papilla stents had fewer episodes of pancreatitis and hospitalizations [59]. The current practice is minor papilla sphincterotomy (miES) with stenting of the dorsal duct. This practice remains to be validated by randomized studies. The SHARP trial is a randomized controlled trial that will determine whether ERCP with minor papilla ES benefits patients with idiopathic RAP and pancreas divisum [60].

### 3.13. Pancreatic Fluid Collection and Duct Leak

**Figure 8:**
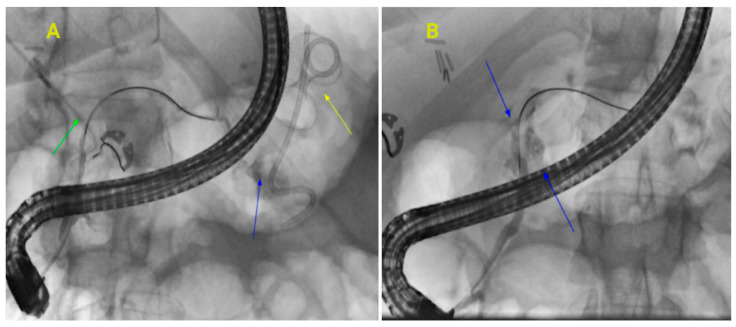
(**A**): Peripancreatic fluid collection treated with LAMS cystogastrostomy as well as ERCP with transpapillary PD stent. Disrupted duct in the tail of the pancreas leading into the pseudocyst cavity. (Green arrow—post cholecystectomy abdominal drain, blue arrow—extravasation of contrast from the tail of PD into the cyst cavity, yellow arrow—Cystgastrostomy using LAMS). (**B**): A tail leak and multiple side branches leaking on pancreatogram concerning disrupted duct (Blue arrows—indicate leakage from multiple side branches).

Pancreatic duct (PD) leaks occur in the setting of acute pancreatitis causing ductal disruption. Low-grade leaks often are asymptomatic or can result in mild symptoms. High-grade leaks are associated with peripancreatic fluid collections (PFC), abdominal fluid collections such as pancreatic ascites, or high amylase pleural effusions. Endoscopic therapy is warranted if large asymptomatic fluid collections persist despite conservative management, as well as those that cause symptoms secondary to compression of contiguous organs, sepsis, fistulization, or persistent pain. Pseudocysts are the most common presentation of a PD leak. The acute collections tend to encapsulate after 4 weeks or more. These collections persist in 30–50% of acute fluid collections. A mature collection can cause gastric or duodenal outlet obstruction. Treatment entails the creation of a fistulous tract between the PFC and the gastrointestinal lumen by the placement of stents. Drainage of PFCs has historically been performed without EUS using a needle knife or cystotome to access the cyst cavity, followed by dilation of the tract and placement of double pigtail stents. A randomized prospective trial [61] showed higher technical success for EUS-guided access (94% vs. 72%) and its utility in cases where there is a lack of bulging in the gastric wall [62] performed a meta-analysis of randomized trials and prospective studies involving 229 patients and confirmed the higher technical success rate of pseudocyst drainage. Since the development of lumen-apposing metal stents (LAMS) by Binmoeller in 2011 [63], their application has revolutionized the management of PFCs. Refer to Figure 8. The large diameter (10–20 mm) which ensures the apposition of two walls and delivery of electrocautery to create a fistulous tract makes it an effective option for drainage of various PFC including pseudocysts and walled-off necrosis (WON). A LAMS needs to be removed in 4 weeks to minimize the risk of bleeding. In contrast, plastic stents can be left indefinitely. Common practice is to replace the LAMS with pigtail stents before the cavity collapses. It is hypothesized that the conformation of the intracavitary flange of the Hot Axios with rigid spikes can lead to trauma of the back wall of the cavity as it shrinks. The advent of another LAMS in 2016, the Spaxus stent which has rounded edges and flanges that fold back, theoretically reduces this risk of bleeding. This prosthesis is not available in the United States. A propensity-matched study showed that bleeding requiring transfusion occurred more commonly in the Hot Axios group than in the Spaxus group. (6.8% vs. 1.5%) [64]. The lumen of the LAMS is enlarged to allow the endoscope to perform necrosectomy. A LAMS with a diameter of 15 or 20 mm is preferred for placement into walled-off necroses. Repeat endoscopies are needed to debride the necrotic material within the cavity. Accessories such as snares, forceps, and Roth nets have been traditionally used for direct endoscopic necrosectomy [65]. A novel device known as EndoRotor (Microtech) allows for non-thermal debridement, combining the functions of suction, irrigation, and tissue dissection for symptomatic WON [66].

### 3.14. Chronic Pancreatitis

Abdominal pain is the most common symptom experienced by patients with chronic pancreatitis (CP). The causes of this pain are complex and often related to factors such as strictures, gallstones, or fluid collections around the pancreas. The primary goal of endotherapy is to manage pain and address complications associated with CP. Traditionally, computed tomography (CT) and magnetic resonance imaging (MRI) have been used for diagnosing CP, along with clinical evaluations. However, endoscopic ultrasound (EUS) is increasingly utilized because it provides detailed structural information about pancreatic tissue and helps evaluate ductal issues. Endoscopic management of chronic pancreatitis involves decompressing the pancreatic duct by dilating strictures or performing lithotripsy to reduce the stone burden. Assessing both the stone burden and the location of the calculi, whether they are intraductal or within the pancreatic tissue, can aid in selecting the appropriate treatment approach. Endoscopic retrograde pancreatography (ERP) with PD stenting may be offered for patients with pancreatic duct (PD) strictures. Removing PD stones can alleviate pain in symptomatic patients and may potentially help to preserve gland function in asymptomatic patients; however, the latter is not routinely recommended due to insufficient clinical data. Patients with multifocal strictures or extensive parenchymal calcifications may need to be referred for surgical management. Most patients with CP are treated using an initial trial of endotherapy prior to referral for surgery. However, three randomized trials from Europe showed greater improvement in pain in the early surgery group compared to endoscopic management [67,68,69]. Despite the available evidence, endotherapy remains the initial approach in most centers given its less invasive nature and the perception of lower risk compared with surgery. It is a reasonable alternative to surgery in candidates with significant comorbidities. An ideal candidate for endotherapy is those with single dominant stricture or an obstructive calculus in the head.

### 3.15. Endoscopic Management of PD Calculi and Strictures

**Figure 9:**
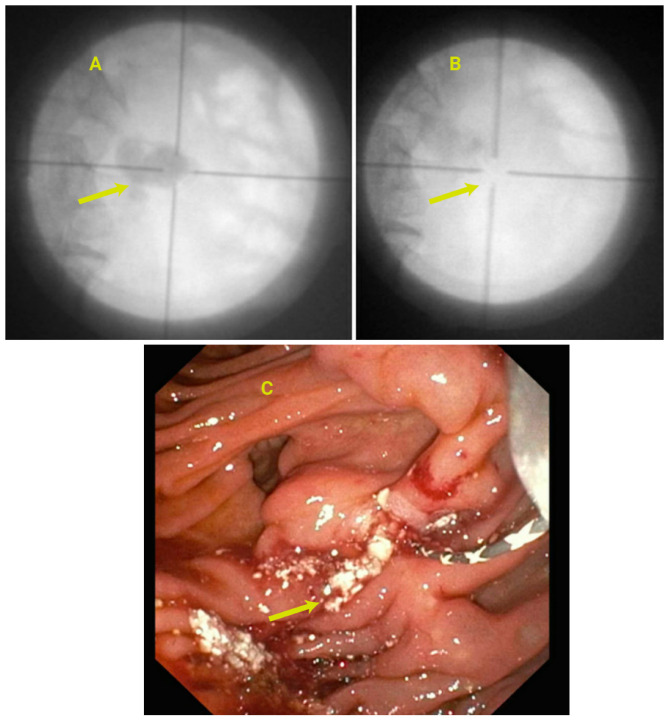
(**A**): Extracorporeal shock wave lithotripsy, 5000 shock waves performed targeting the stone. (**B**): Fragmentation of the radiopaque stone. (**C**): ERCP with stone retrieval. Arrow—fragmented stones.

ERCP can be attempted to clear stones using an extraction balloon or stone basket. The success of clearance depends on the size of the stone, the presence of strictures, and the degree of impaction within the duct. A small pancreatic sphincterotomy is performed to allow the removal of stones and to improve drainage. Various anatomical variations of the main PD such as ansa pancreaticus or partial ansa loops can make passage of ERCP accessories challenging. Extracorporeal shock wave lithotripsy (ESWL) is recommended for stones larger than 5 mm to achieve fragmentation. ESWL can be used to fragment large stones or those adjacent to a stricture, followed by ERCP to achieve ductal clearance. Refer to Figure 9. ESWL monotherapy may provide pain relief for selected patients suffering from painful chronic pancreatitis with main pancreatic duct (PD) obstruction [70]. A meta-analysis of 22 studies found that complete ductal clearance was achieved in 70% of cases, and pain relief was reported in 64%. Stone fragmentation occurred in 86% of the patients. However, adverse events, including pancreatitis and cholangitis, were noted in 4% of the cases [71].

ESWL is contraindicated for patients with coagulopathy or calcified blood vessels in the path of the shock waves. An effective alternative treatment is peroral pancreatoscopy-assisted electrohydrolithitripsy (POP EHL) or laser lithotripsy (LL). A study by Atwell et al., involving 46 patients with PD calculi, demonstrated complete ductal clearance in 70% of participants [72]. Furthermore, a systematic review of 10 studies on POP with lithotripsy indicated that the pooled technical success rate was 92%, with a fragmentation success rate of 86% [73]. The potential advantages of POP-guided lithotripsy include direct visualization and targeting of stones. Adverse events include PEP (7%), perforation, or hemorrhage (3–4%) [74].

In cases with PD stricture, an EUS should be performed to exclude pancreatic malignancy. The views during EUS can be challenging due to the shadowing of calculi. Additional imaging, such as pancreas protocol CT or MRCP, can also be performed. Endoscopic treatment of PD strictures involves performing a pancreatic sphincterotomy and dilation of the stricture using a balloon or a dilating catheter. One or more stents are placed routinely and replaced every 4–6 months. Endotherapy should be performed every 12 months for stricture remodeling. Refractory strictures can be treated with multiple plastic stents or FCSEMS [75,76]. Adverse events reported are worsening of pain, stent migration, pancreatitis, or cholangitis. Pain is associated with the placement of larger stents over 8 mm in diameter [77].

Biliary strictures develop in 10–15% of patients with CP [78]. Similar to other strictures, treatment with multiple plastic stents or SEMS should be considered. In a long-term follow-up study, 62% of patients were stent-free after an FCSEMS after 5 years. However, severe CP and a longer length of stricture are associated with stricture recurrence [79].

### 3.16. Role of EUS in the Management of CP

EUS is an important diagnostic and therapeutic tool in the management of CP. Peripancreatic fluid collections occur in patients with CP. EUS-guided drainage is preferable with or without concomitant transpapillary PD stent placement if an active ductal leak can be bridged with the prosthesis. When drainage of the PD cannot be achieved by other methods, EUS-guided pancreaticogastrostomy can be performed in patients who are poor candidates for surgery or with post-surgical anatomy [80]. Celiac plexus blockade can be considered for pain management. The celiac ganglia are located anterolateral to the aorta, with the right ganglion positioned 6 mm inferior and the left ganglion 9 mm inferior to the origin of the celiac artery. It is important to note that the celiac ganglion may not be visible as a distinct structure during EUS. Celiac plexus blockade involves the injection of a local anesthetic and corticosteroids adjacent to and anterior to the lateral aspect of the aorta under direct visualization. The most serious adverse event associated with this procedure is the accidental injection into the spinal artery, which can lead to paraplegia. This risk is primarily associated with classic or posterior approach block techniques performed percutaneously [81]. Additionally, there is a risk of pneumothorax if there is a transverse entry through the diaphragm, a situation that can occur with the posterior approach. It is also worth noting that diarrhea has been reported as well as a mild decrease in mean blood pressure.

## 4. Conclusions

Endoscopic management has become a cornerstone in treating benign pancreaticobiliary disorders, offering minimally invasive alternatives for patients who are poor surgical candidates or present with complex anatomical challenges. Techniques such as ERCP, ETP-GBD, EUS-GBD, and advanced lithotripsy methods allow for effective intervention in conditions like acute cholecystitis, choledocholithiasis, chronic pancreatitis, and post-operative bile duct strictures and bile leaks. Innovations in stent technology, including lumen-apposing metal stents (LAMS) and fully covered self-expanding metal stents (FCSEMS), have improved technical success and clinical outcomes while minimizing risks. Additionally, EUS plays a pivotal role in diagnostics and therapeutic interventions. The minimally invasive nature of these endoscopic techniques provides patients with reduced recovery times and lower complication rates compared to surgery. However, these approaches demand procedural expertise and individualized assessment. As technology and techniques advance, endoscopic management continues to expand the range of safe, effective treatment options which may include magnetic compression anastomoses, the integration of artificial intelligence, and robotic-assisted endoscopy, further underscoring its value as an essential therapeutic approach in benign pancreaticobiliary diseases.

## Figures and Tables

**Table 1:** Selected studies on technical and clinical outcomes of transpapillary gallbladder drainage (ETP-GBD).

Study	No. of Patients	Technical Success (%)	Clinical Success (%)	Adverse Events (%)	Need for Recurrent Intervention (%)
Oh et al. [12]	96	86.6	86	19.3	10.4
Higa et al. [13]	38	87.2	76.3	9.4	15.8
Siddiqui et al. [14]	124	87.9	79.8	7.3	11.4
Inoue et al. [15]	90	78.6	74.4	21.1	Not reported

**Table 2:** Technical success rates of endoscopic ultrasound-guided gallbladder drainage.

Study	N	Technical Success %	Clinical Success %	Adverse Events %	Need for Recurrent Interventions %
Tyberg et al. [20]	42	95.2	95.2	21.4	11.9
Irani et al. [3]	45	97.8	95.6	17.8	24.4
Teoh et al. [4]	39	97.4	92.3	12.8	2.6
Jang et al. [5]	30	96.7	96.7	6.7	-
Oh et al. [12]	76	99.3	99.3	7.1	3.9
Higa et al. [13]	40	97.5	95	17.9	17.9
Siddiqui et al. [14]	102	94.1	90.2	11.8	0
Inoue et al. [15]	90	96.7	88.9	12.2	-

**Table 3:** Outcomes in liver transplant anastomotic strictures using plastic stents.

	N	Technical Success	Stricture Resolution	Stricture Recurrence Rate
Rerknimitr et al. [52]	43	100	100	0
J.Morelli et al. [53]	25	96	88	9
Alazmi et al. [54]	148	97	89	18
Pasha et al. [55]	25	100	72	22
Holt et al. [56]	53	92	64	3
G Morelli et al. [57]	38	100	89	15
Tabibian et al. [58]	69	100	94	3

## Data Availability

Not applicable.
